# Potentially Overestimated Efficacy of Nanoparticle Albumin-bound Paclitaxel compared with Solvent-based Paclitaxel in Breast Cancer: A Systemic Review and Meta-analysis

**DOI:** 10.7150/jca.59794

**Published:** 2021-06-22

**Authors:** Bing-Xue Li, Xin-Jie Chen, Tong-Jing Ding, Yi-Hua Liu, Ting-Ting Ma, Gan-Lin Zhang, Xiao-Min Wang

**Affiliations:** 1 Department of Oncology, Beijing Hospital of Traditional Chinese Medicine, Capital Medical University, No.23 Gallery Back Street, Dongcheng District, Beijing, 100010, PR China.; 2 Beijing University of Chinese Medicine, No.11 East Road, North of the Third Ring, Chaoyang District, 100029, PR China.

**Keywords:** breast cancer, nab-paclitaxel, paclitaxel, efficacy, toxicity, meta-analysis

## Abstract

**Background:** Nanoparticle albumin-bound paclitaxel (nab-PTX) has exhibited clinical efficacy in breast cancer treatment, but toxicities can be yielded more at the same time. We did this meta-analysis aiming to unambiguously compare nab-PTX with conventional solvent-based paclitaxel (sb-PTX) in breast cancer patients of all stages.

**Method:** Pubmed, Embase and Cochrane Library were searched for head-to-head randomized controlled trials of nab-PTX and sb-PTX in breast cancer. Risk ratio (RR) with 95% confidence interval was used for dichotomous variables while Hazard ratio (HR) was used for time-to-event outcomes.

**Results:** Our review finally included 9 studies with 3508 patients. Nab-PTX showed a benefit on objective response rate (ORR) (RR=1.22 [1.04-1.43], P=0.01) as well as non-inferiority compared with sb-PTX in disease control rate (DCR) (RR=1.01 [0.98-1.04], P=0.44), overall survival (OS) (HR=0.99 [0.93-1.05], P=0.81) and disease free survival/progression free survival (DFS/PFS) (HR=0.92 [0.81-1.05], P=0.21). However, when it comes to toxicities (fatigue, nausea or vomiting, peripheral sensory neuropathy and adverse event related discontinuation), results favored sb-PTX (RR=2.89 [1.07-7.8], 3.15 [1.78-5.59], 2.11 [1.32-3.37], 2.02 [1.61-2.53]; P<0.05). Patients with metastatic tumors or undergoing conventional schedule responses better to nab-PTX than the compared groups (RR of ORR in metastatic vs early or locally advanced patients: 1.46 [1.09-1.96] vs 1.01 [0.94-1.08]; conventional vs dose dense group: 1.59 [1.23-2.06] vs 1.01 [0.91-1.12]).

**Conclusions:** Nab-PTX can improve ORR compared with paclitaxel and should be given priority to when aiming to reduce tumor load in breast cancer. Sb-PTX of dose dense schedule is recommended when toxicity of nab-PTX is hard to bear for breast cancer patients.

## Introduction

There were 2,261,419 new cases of breast cancer, ranking first among all cancer types worldwide, according to the data of GLOBOCAN 2020 1. Chemotherapy is the method mostly commonly used in all treatment regimens including neoadjuvant treatment for inoperable or locally advanced cancer patients, adjuvant treatment for patients of early stage after surgery with high risk of recurrence, and systemic treatment for recurrent/stage IV disease in breast cancer. Except for stage I or part of stage II estrogen receptor positive (ER+)/human epidermal growth factor receptor 2 negative (Her2-) breast cancer patients, nearly all other breast cancer patients should undergo chemotherapy to gain a better prognosis 2-4. Among all the agents for chemotherapy, taxanes, targeting microtubules by promoting polymerization and stabilization of tubulin and thus interfering with the mitotic spindle 5-6, firstly found in 1971, stand out for the anti-cancer effect and has been approved to treat breast cancer by FDA since 1994. Trials looking for newer cytotoxic agents to treat breast cancer have not yet identified any classes that are clearly superior to taxanes and anthracyclines 7-9. Considering the great popularity of taxanes and the development of precision medicine, digging into a more effective using method of taxanes together with less toxicity in breast cancer is necessary and feasible nowadays.

There are different forms of taxanes frequently used in clinical practice in breast cancer, including solvent-based paclitaxel (sb-PTX), docetaxel (a semi-synthetic analog of paclitaxel) and nanoparticle albumin-bound paclitaxel (nab-PTX), three of which are most frequently used worldwide. Sb-PTX is the first to be identified and should be dissolved in Cremophor EL which may lead to hypersensitivity reactions and make dexamethasone and H1 & H2 -receptor antagonists necessary as premedication. Nab-PTX, a solvent-free nanometersized form of paclitaxel which make castor oil caused toxicity avoidable, can be administered with shorter fusion schedule (30 minutes) than sb-PTX and no premedication is needed 10-11. Even though these two drugs show different traits in distribution, clearance, systemic exposure as well as transportation to tumors and tumor uptake of PTX 12, they use the same effective constituent while docetaxel does not and thus docetaxel has unique characteristics like higher affinity with tubulin leading to higher anti-cancer effect and different toxicity profile. In places like China, with the decreased price caused by development of generic nab-PTXs and the easy-to-use peculiarity, nab-PTX prevails among the three. Taking the features of each drug and the potential overuse of nab-PTX into consideration, and with the aim to see the pure effect induced by administration form other than drug's chemical structure, comprehensive comparison of sb-PTX and nab-PTX is needed.

There are three meta-analyses 13-16 which compared nab-PTX with sb-PTX in the subgroup analysis, while one 15 has mixed sb-PTX with docetaxel up and no such subgroup analysis was done. Among the former three, two studies 13-14 regarding neoadjuvant chemotherapy included retrospective trials or cohort trials and therefore lack credibility. The remaining one only included two trials 10,17 and omitted one important study of high recruitment 18 with no reason supplied. What's more, trial providing toxicity outcomes only 19 were not included even in the toxicity related comparisons. In sum, no overview of the two drugs' efficacy and toxicity has been fully unfolded.

To help make a wide choice between nab-PTX and sb-PTX, we performed this meta-analysis of data from all the head-to-head randomized controlled trials comparing nab-PTX and sb-PTX in breast cancer regardless of treatment settings. The following outcomes were compared: short term efficacy (objective response rate, disease control rate), long term efficacy (overall survival, DFS in neoadjuvant treatment or PFS in metastatic disease) and toxicity (≥3 grade toxicity events and adverse effect related discontinuation). Subgroup analyses were done using available data and treatment settings/schedule/molecular subtype were all taken into account.

## Methods

### Literature search strategy

This meta-analysis was done following recommendations of the Preferred Reporting Items for Systematic Review and meta-analysis (PRISMA) 20. Pubmed, Embase and Cochrane Library were searched for relevant studies published before 23 January, 2021. The major search terms were ('breast neoplasms' or 'breast cancer' or 'breast tumors') and ('albumin-bound paclitaxel' or 'Abraxane' or 'ABI 007') and ('paclitaxel' or 'Taxol' or 'NSC125973'). Detailed search methods could be get in Supplement File. Abstracts from American Society of Clinical Oncology (ASCO), Chinese Society of Clinical Oncology (CSCO) and European Society for Medical Oncology (ESMO) and San Antonio Breast Cancer Symposium (SABCS) were also retrieved using similar search terms for supplementation.

### Study selection

We only included randomized clinical trials comparing nab-PTX with sb-PTX in this study. Observational studies, case reports, animal studies, those with unclear data on outcomes or comparing nab-PTX with docetaxel, were all excluded. The selecting process was independently conducted by two authors (Bing-Xue Li & Yi-Hua Liu). Any disagreements between these two investigators were resolved by a third author (Xin-Jie Chen).

### Data extraction and quality assessment

We extracted data on study randomization methods, participants' baseline characteristics, chemotherapy regimens and outcomes together with outcome definitions both in printed papers and electronic files (excel). Outcomes were measured as follows: 1) objective tumor response rate (ORR, the proportion of participants with a complete or partial response); 2) disease control rate (DCR, the proportion of participants with a complete response, partial response or stable disease); 3) overall survival (OS, time from date of randomization to date of death caused by any cause); 4) progression-free survival/disease-free survival (PFS/DFS, the time for date of randomization to date of progression or death caused by any cause); 5) grade 3/4 toxic events as defined by the original study. Two investigators (Bing-Xue Li & Xin-Jie Chen) independently extracted the data and resolved queries through discussion with a third researcher (Tong-Jing Ding). For trials with three arms including nab-PTX and sb-PTX, we only extracted data of this two arms. When there was more than one publication on the same study, we used the updated data for the long-time follow-up outcomes. As for missing data, attempts have been made to contact the original investigators but further details were not available and these data were excluded from the quantitative analysis.

The Cochrane Collaboration's 'Risk of bias' assessment tool 21 was used to assess the potential source of bias in the included studies. Three personnel (Bing-Xue Li, Xin-Jie Chen, Tong-Jing Ding) did the assessment independently and the result of another meta-analysis with published risk of bias summary 15 was also referred to. When there were unsolvable conflicts, a forth investigator (Yi-Hua Liu) was asked to give the final judgement.

### Statistical analysis

Risk ratio (RR) with 95% confidence interval were used for dichotomous variables while hazard ratio (HR) for time-to-event outcomes. When HRs and SEs were not directly reported in the original paper, we used Engauge Digitizer to obtain the survival rate at different time points from Kaplan-Meier curves and calculated HRs and SEs using the spreadsheet shared by Jayne F Tierney 22. Fixed effect model was used when I^2^<50% and p>0.1 for Q test otherwise random effects model was used (outcome of two methods were all shown in the forest plot but the result of the right model was indicated by the purple diamond). Heterogeneity was measured by I^2^ and explored by subgroup analysis, baujat plot and sensitivity analysis (omitting each study once at a time). In tests of subgroup differences, fixed effect model was only used when there was no heterogeneity found in all the subgroups or overall results, otherwise random effects model would be used. Publication bias was evaluated by funnel plots and adjusted using trim-and-fill method. All the analyses were done using R version 4.0.2.

## Results

### Description of included studies

**Figure 1:**
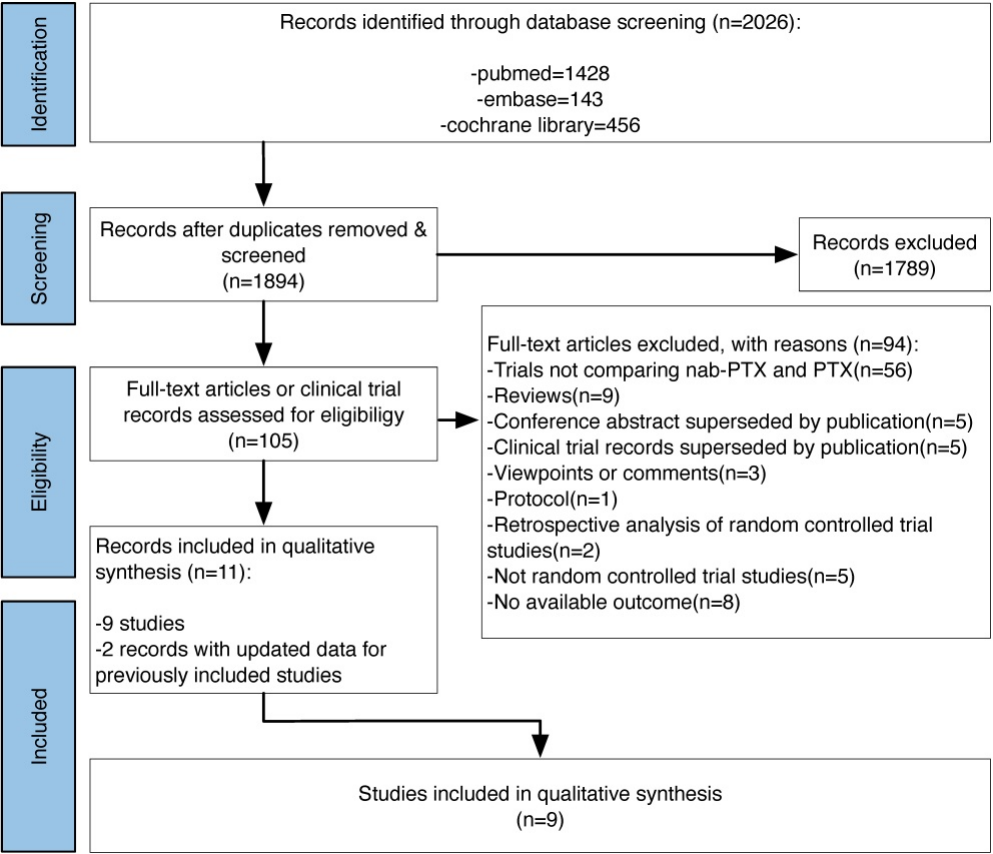
Study flow diagram.

After searching pubmed, embase and the cochrane library, we got 2026 records on January 23 2021. Finally we contained 11 records [10,17-18,23-30], 2 [24,28] of which provided updated data for 2 previously included studies. The study flow diagram can be seen in Figure 1.

Overall, 3508 patients were included in the analysis. For trials including more than two arms 18-19,28, we only extracted data of the nab-PTX and sb-PTX groups in our meta-analysis. Main characteristics of the included studies can be seen in Table 1.

### Risk of Bias of the Included Studies

For selection bias, 6 trials 10,17-18,26-29 were at unclear risk of allocation concealment because no related information was available.

For performance bias, considering the shorter fusion schedule (30 minutes) and no premedication in nab-PTX group, there was no possible way to blind the participants and investigators, so we assessed all studies at high risk.

Detection bias was grouped by outcomes with similar risk of bias:

**1) ORR, DCR, PFS/DFS:**

Untch 2016^23^-^24^ and Rugo 2015^18^,^28^ were at unclear risk because the criteria of tumor response evaluation was not mentioned in the original paper.

**2) OS, hematological toxicity:**

We perceived that these outcomes' assessments were unlikely to be influenced by blinding or not so we assessed them as at low risk of bias.

**3) Non-hematological toxicity:**

No details were provided so we assessed them as at unclear risk.

We drew the conclusion that time-to-event outcomes (OS and PFS) were all incomplete because the specific censor method (like if people changed to another anti-cancer drug, they were assigned as censored or still in trial?) was not explained and this would have an effect on the analyzed result. Ciruelos 2019 19 was not evaluated because it only reported 5-year survival rate and did not provide time-to-event outcomes.

Considering the baseline characteristics of the included studies differed and the potential factors influencing the outcomes were not fully understood, all studies was assessed as at unclear sick of bias in the 'other bias' part.

**Figure 2:**
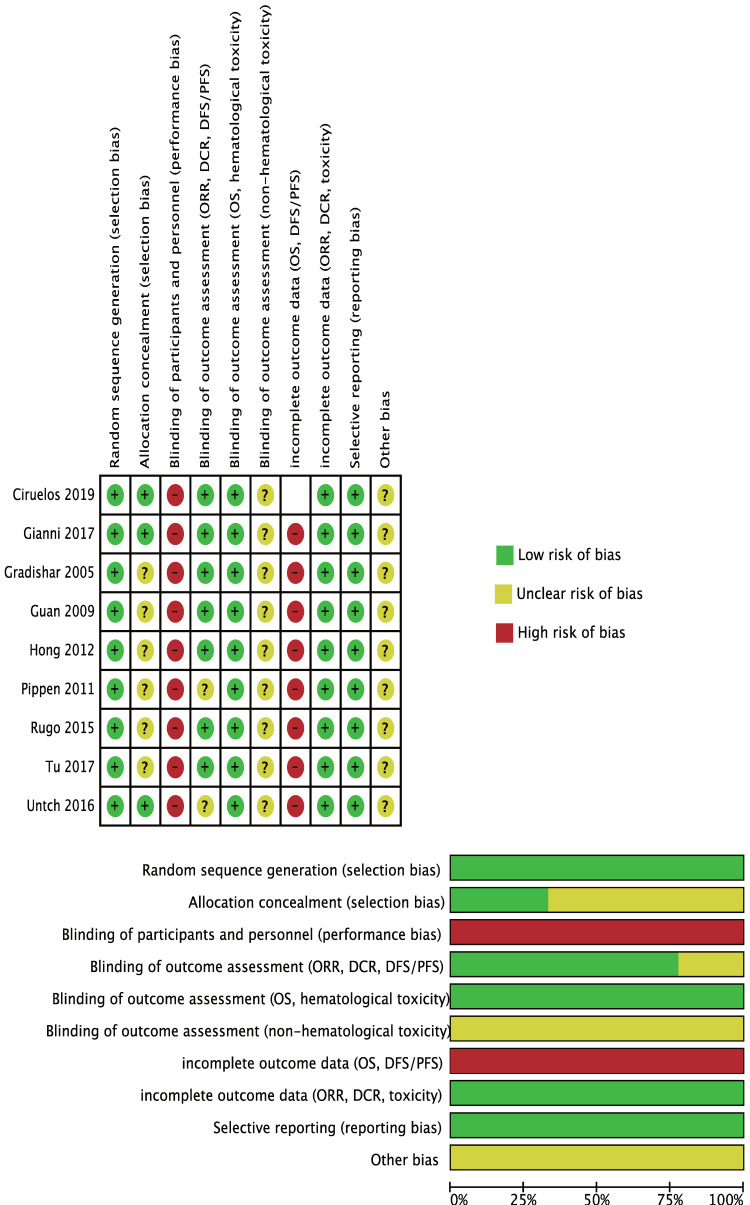
Risk of Bias.

All the quality assessment could be referred in Figure 2.

## Analysis Results

### Overall analysis

#### The efficacy of nab-PTX vs sb-PTX

**Figure 3:**
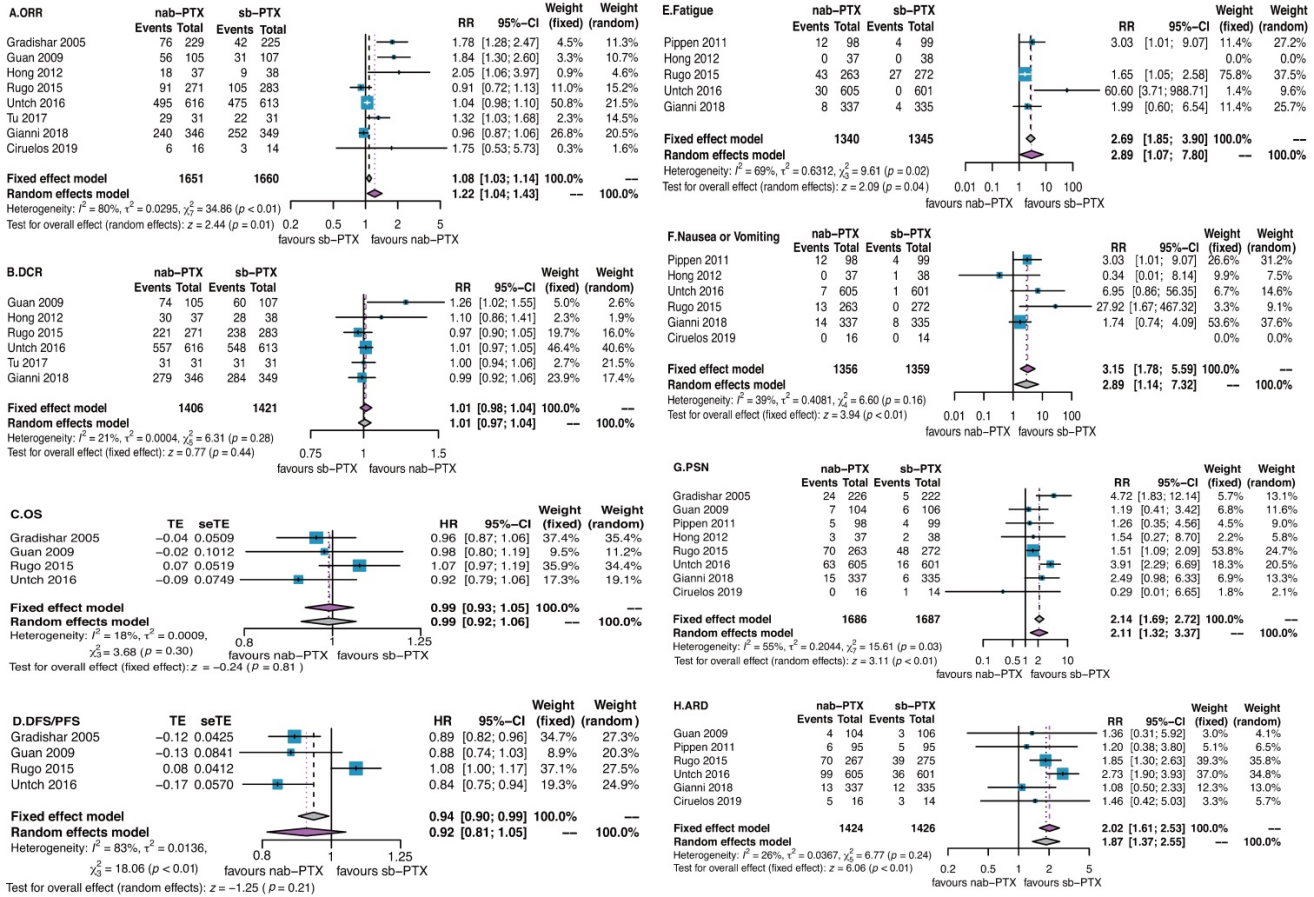
** Overall analyses of all the efficacy and toxicity outcomes.** Significant difference was found in analysis of objective response rate in favor of nab-PTX (P=0.01) while it was on the contrary in toxicities as fatigue, nausea or vomiting, peripheral sensory neuropathy, and adverse event related discontinuation (P<0.05). Abbreviations: ORR, objective response rate; DCR, disease control rate; OS, overall survival; DFS/PFS, disease-free survival/progression-free survival; PSN, peripheral sensory neuropathy; ARD, adverse event related discontinuation.

Eight studies were available for us to estimate a risk ratio (RR) for objective response rate (ORR). Specially, for patients undergoing neoadjuvant chemotherapy, we analyzed the response rate before surgery other than pathological complete response rate (pCR) to evaluate the pure effect of chemotherapy. The ORRs were respectively 61.2% in nab-PTX group and 57% in sb-PTX group. There was a significant difference in favor of nab-PTX with an RR of 1.22 (95% CI 1.04 to 1.43; P=0.01; Participants = 3311; Studies = 8; Figure 3A). No significant differences were found in the analyses of disease control rate (DCR, Figure 3B), overall survival (OS, Figure 3C), disease-free survival/progression-free survival (DFS/PFS, Figure 3D).

#### The toxicity of nab-PTX vs sb-PTX

We analyzed 11 toxicity events (anaemia, leucopenia, thrombopenia, neutropenia, febrile neutropenia, fatigue, nausea or vomiting, peripheral sensory neuropathy, adverse-event related discontinuation) which were reported by more than 2 studies. Differences were found in fatigue (nab-PTX vs sb-PTX: 7% vs 3%), nausea or vomiting (nab-PTX vs sb-PTX: 3% vs 1%), peripheral sensory neuropathy (nab-PTX vs sb-PTX: 11% vs 5%), adverse event related discontinuation (nab-PTX vs sb-PTX: 14% vs 7%) (Figure 3E-H).

### Overall heterogeneity

Overall statistical heterogeneity was found in ORR (I^2^=80%, P<0.01), DFS/PFS (I^2^=83%, P<0.01), fatigue (I^2^=69%, P=0.02) and PSN (I^2^=55%, P=0.03) while not found in DCR (I^2^=21%, P>0.05), OS (I^2^=18%, P>0.05), nausea or vomiting (I^2^=39%, P>0.05) and ARD (I^2^=26%, P>0.05) (Figure 3).

### Subgroup analysis

Statistical subgroup differences were only found in analysis of ORR between different treatment settings (P=0.02) and different schedules (P<0.01). Patients with metastatic tumors or undergoing conventional schedule responded better to nab-PTX than the compared groups (RR of ORR in metastatic vs early or locally advanced patients: 1.46 [1.09-1.96] vs 1.01 [0.94-1.08]; conventional vs dose dense group: 1.59 [1.23-2.06] vs 1.01 [0.91-1.12]; Figure S1-8 subgroup analysis 1-2). However, in dose dense subgroup analyses, toxicities occurred more frequently in patients treated with nab-PTX (RR of fatigue in dose dense group 2.89 [1.07,7.80], nausea or vomiting 3.15 [1.78,5.59], PSN 2.00 [1.17,3.42], ADR 2.04 [1.62, 2.57]; Figure S5-8 subgroup analysis 2).

Molecular subtype outcomes were only available for triple negative breast cancer (TNBC) in OS and DFS/PFS analyses but still no significant difference was found between nab-PTX and sb-PTX (HR of OS in TNBC: 0.88[0.76-1.01], P=0.06; HR of DFS/PFS in TNBC: 0.89 [0.79-1.01], P=0.37). However, HRs in TNBC tended to be lower than that of the total (OS 0.88 vs 0.99; DFS/PFS 0.89 vs 0.92; Figure S3-4 subgroup analysis 3).

### Sensitivity analysis

Among all the top 2 studies which influenced outcome the most when omitted, Rugo 2015 ranked first while Gianni 2018 and Untch 2016 ranked second. Rugo 2015 influenced DCR, OS, DFS/PFS, fatigue, nausea or vomiting, PSN greatly; except for nausea or vomiting, the other five outcomes were also verified by baujat plot. Gianni 2018 influenced ORR, DCR, nausea or vomiting, and ARD and two of the outcomes (ORR, nausea or vomiting) were verified by baujat plot. Untch 2016 affected ORR, fatigue, PSN and ARD and only ADR was verified by baujat plot. Related results could be got in Sensitivity analysis & Baujat plot parts of Figure S1-8.

Based on Q test results, for ORR and DCR, Guan 2009 contributed most to heterogeneity; for OS, DFS/PFS, nausea or vomiting, Rugo 2015 contributed the most; for fatigue, PSN and ADR, Untch 2016 contributed the most (Baujat plot parts of Figure S1-8).

### Publication bias

Funnel plots showed that publication bias might exist in all the analyses. And after using trim-and-fill method, RR of ORR was still higher than 1.00 and RRs of fatigue and nausea or vomiting became lower than before adjusted, which encourage the usage of nab-PTX. But HRs for OS and DFS/PFS became higher which discourage the usage of nab-PTX.

## Discussion

We tended to compare nab-PTX with sb-PTX comprehensively in breast cancer patients of all stages in our study. We have confirmed the superiority in objective response to nab-PTX compared with sb-PTX but with higher occurrence of fatigue, nausea or vomiting, peripheral sensory neuropathy and adverse event related discontinuation, while it showed non-inferiority in DCR, OS and DFS/PFS.

Unlike previous meta-analyses 13-16, we have restricted the comparison group as only sb-PTX instead of sb-taxanes (sb-PTX and docetaxel) to exclude the influence of docetaxel which is of different chemical structure with the other two taxanes. But as related studies are limited, we have also enlarged our scope of included study to all stages of breast cancer to increase our sample size as possible as we can. Subgroup analyses considering stages were done at the same time to avoid the influence of different treatment regimens. However, ORR results still favors nab-PTX in patients with metastasis disease which are in line with previous studies. But RR for ORR of patients undergoing neoadjuvant chemotherapy (i.e. early or locally advanced patients) was not significant (Figure S1) which differed from other studies 13-14. The reason might lie in the following 3 reasons: 1) we used ORR rather than pCR to evaluated the effect; 2) docetaxel, which was included in their studies, has different chemical structure and toxicity profile, and could make the result more confusing; 3) these studies have also included observational studies. Thus our result might be more credible but more studies still need adding to verify the result. As previous studies 30-31 have demonstrated that dose dense schedule outweighs conventional schedule regimen, we added subgroup analyses of treatment schedules. Patients in conventional schedule group responded better to nab-PTX than to sb-PTX, while in dose dense group no significant difference was found. This indicated that even though nab-PTX outperformed sb-PTX in tumor uptakes of PTX, adjusting the schedule into dose dense may make up for the difference in efficacy. Considering the data for ORR in our study and the establishment of Gompertzian model of tumor growth^32^ as well as related meta-analysis of chemotherapy studies in breast cancer^33^, we now ranked the anti-cancer efficacy of the two drugs in two dose intensities as dose dense nab-PTX ≥ dose dense sb-PTX > conventional nab-PTX > conventional sb-PTX.

No obvious advantage in DCR, OS and DFS/PFS was seen in nab-PTX in our study. But taking the higher adverse event related discontinuation in nab-PTX group into account, time-to-event outcomes might have been overestimated as censoring method in survival analysis could be informative and non-independent 34. And the non-inferiority of nab-PTX comparing with PTX in overall survival could not be firmly believed. As is with the DFS/PFS result. More scientific analysis method of survival is needed to see the actual effect of cytotoxic drugs. What's more, publication bias might exist in OS and DFS/PFS. More studies with long-time follow-up are need to get a more accurate result.

Results of TNBC group in OS and DFS/PFS still showed a trend to favor nab-PTX but no statistical significance was found. With the development of precision medicine, individual patient data with more molecular information might help dig deeper into the benefit population of nab-PTX in the future. SPARC 35-37, could also be further evaluated as a predictive marker especially for response to nab-paclitaxel, but additional concerns about the status of SPARC and its cut-off value are still certainly needed in clinical trial design, statistical analysis and outcome interpretation.

For toxicity, risks at least doubled in analyses of fatigue (Figure 3E), nausea or vomiting (Figure 3F), peripheral sensory neuropathy (Figure 3G) and adverse event related discontinuation (Figure 3H). These toxicities should be taken seriously with and could not be ignored in clinical practice.

Just as Lee et al. mentioned 16 and the US Food and Drug Administration (FDA)'s withdrawn approval of bevacizumab in combination with paclitaxel in the treatment of MBC in December 2010, we also found that the addition of bevacizumab to paclitaxel had greatly influenced the final result of ORR, DCR, OS and PFS/DFS and the combination of bevacizumab was not recommended according to these evidence though it was still approved in some other countries.

All in all, the toxicity of nab-PTX should be taken seriously in clinical practice. Apart from publications bias, censoring method could also cause bias in efficacy evaluation of survival analysis which should be considered in further studies. And based on our review, nab-PTX outperformed sb-PTX in objective response rate and should be preferred in conditions when it is urgent to shrink the tumor burden. But nab-PTX can increase risks of fatigue, nausea or vomiting, peripheral sensory neuropathy and adverse-related discontinuation at the same time. The difference between nab-PTX and sb-PTX in dose dense group was not obvious, so when toxicity of dose dense scheduled nab-PTX are unbearable, change it directly into sb-PTX is ponderable.

## Supplementary Material

Supplementary methods and figures.

## Figures and Tables

**Table 1:** Characteristics of the included studies

Study ID	Setting	Regimen	Drug	ITT	Actual Participants	Dose	Schedule	Cycles
Untch 2016 23,24	neoadjuvant	ddT(HP)→EC(HP)	nab-PTX	616	605	150mg/m^2^→125mg/m^2^	d1, 8, 15 q3W	4
			sb-PTX	613	601	80mg/m^2^	d1, 8, 15 q3W	
Gianni 2018 25	neoadjuvant	ddT→AC/EC/FEC	nab-PTX	346	337	125mg/m^2^	d1, 8, 15 q4W	4
			sb-PTX	349	335	90mg/m^2^	d1, 8, 15 q4W	
Pippen 2011 26	adjuvant	ddAC→TB	nab-PTX	98	95	260mg/m^2^	d1 q2W	4
			sb-PTX	99	95	175mg/m^2^	d1 q2W	
Gradishar 2005 10	metastatic	T	nab-PTX	229	229	260mg/m^2^	d1 q3W	NR
			sb-PTX	225	225	175mg/m^2^	d1 q3W	
Guan 2009 17	metastatic	T	nab-PTX	105	104	260mg/m^2^	d1 q3W	NR
			sb-PTX	107	106	175mg/m^2^	d1 q3W	
Hong 2012 27	metastatic	ddT	nab-PTX	37	37	150mg/m^2^	d1, 8, 15 q4W	4
			sb-PTX	38	38	85mg/m^2^	d1, 8, 15 q4W	
Rugo 2015 18,28	metastatic	ddTB	nab-PTX	271	267	150mg/m^2^	d1, 8,15 q4W	NR
			sb-PTX	283	275	90mg/m^2^	d1, 8, 15 q4W	
Tu 2017 29	metastatic	TE	nab-PTX	31	31	260mg/m^2^	d1 q3W	NR
			sb-PTX	31	31	175mg/m^2^	d1 q3W	
Ciruelos 2019 19*	metastatic	ddT	nab-PTX	16	16	100mg/m^2^	d1, 8, 15 q4W	7
			sb-PTX	14	14	80mg/m^2^	d1, 8, 15 q4W	6

A: doxorubicin; B: Bevacizumab; C: cyclophosphamide; dd: dose dense; E: epirubicin; F: fluorouracil; H: trastuzumab; ITT: intention-to-treat population; NR: not reported; P: pertuzumab; T: taxanes. *In Ciruelos 2019, there were two more groups using nab-PTX but of different schedule from that of sb-PTX so we didn't include this two groups.

## Abbreviations

A: doxorubicin
ARD: adverse event related discontinuation
ASCO: American Society of Clinical Oncology
B: Bevacizumab
C: cyclophosphamide
CSCO: Chinese Society of Clinical Oncology
DCR: disease control rate
dd: dose dense
DFS: disease free survival
E: epirubicin
ER: estrogen receptor
ESMO: European Society for Medical Oncology
F: fluorouracil
H: trastuzumab
Her2: human epidermal growth factor receptor 2
HR: hazard ratio
ITT: intention-to-treat population
nab-PTX: nanoparticle albumin-bound paclitaxel
NR: not reported
ORR: objective response rate
OS: overall survival
P: pertuzumab
pCR: pathological complete response rate
PFS: progression free survival
PRISMA: Preferred Reporting Items for Systematic Review and meta-analysis
RR: risk ratio
SABCS: San Antonio Breast Cancer Symposium
sb-PTX: solvent-based paclitaxel
T: taxanes
TNBC: triple negative breast cancer
