# Supplementary material for: Potentially Overestimated Efficacy of Nanoparticle Albumin-bound Paclitaxel compared with Solvent-based Paclitaxel in Breast Cancer: A Systemic Review and Meta-analysis
Source: J Cancer. 2021 Jun 22;12(17):5164–72. doi: 10.7150/jca.59794 (PMC8317518; doi:10.7150/jca.59794)
Supplement: Supplementary file 1 — Supplementary methods and figures. [file jcav12p5164s1.pdf]

1. PUBMED search strategy

("Breast Neoplasms"[Mesh] OR (Breast Neoplasm[tiab]) OR (Neoplasm, Breast[tiab]) OR (Breast Tumors[tiab]) OR (Breast Tumor[tiab]) OR (Tumor, Breast[tiab]) OR (Tumors, Breast[tiab]) OR (Neoplasms, Breast[tiab]) OR (Breast Carcinoma[tiab]) OR (Breast Carcinomas[tiab]) OR (Carcinoma, Breast[tiab]) OR (Carcinomas, Breast[tiab]) OR (Mammary Neoplasms, Human[tiab]) OR (Human Mammary Neoplasm[tiab]) OR (Human Mammary Neoplasms[tiab]) OR (Neoplasm, Human Mammary[tiab]) OR (Neoplasms, Human Mammary[tiab]) OR (Mammary Neoplasm, Human[tiab]) OR (Breast Cancer[tiab]) OR (Cancer, Breast[tiab]) OR (Mammary Cancer[tiab]) OR (Cancer, Mammary[tiab]) OR (Cancers, Mammary[tiab]) OR (Mammary Cancers[tiab]) OR (Malignant Neoplasm of Breast[tiab]) OR (Breast Malignant Neoplasm[tiab]) OR (Breast Malignant Neoplasms[tiab]) OR (Malignant Tumor of Breast[tiab]) OR (Breast Malignant[tiab]) OR (Breast Malignant Tumors[tiab]) OR (Cancer of Breast[tiab]) OR (Cancer of the Breast[tiab])) AND ("Albumin-Bound Paclitaxel"[Mesh] OR (Albumin Bound Paclitaxel) OR (Paclitaxel, Albumin-Bound) OR (Protein-Bound Paclitaxel) OR (Paclitaxel, Protein-Bound) OR (Protein Bound Paclitaxel) OR Abraxane OR (ABI007) OR (ABI-007) OR (ABI 007)) AND ("Paclitaxel"[Mesh] OR Anzatax OR (NSC-125973) OR (NSC 125973) OR (NSC125973) OR Taxol OR (Taxol A) OR (Bris Taxol) OR (Taxol, Bris) OR (Paclitaxel, (4 alpha)-Isomer) OR (Paxene) OR (Praxel) OR (7-epi-Taxol) OR (7 epi Taxol) OR (Onxol)) AND ((randomized controlled trial[pt] OR controlled clinical trial[pt] OR randomized[tiab] OR placebo[tiab] OR clinical trials as topic[mesh:noexp] OR randomly[tiab] OR trial[ti] NOT (animals[mh] NOT humans [mh])))

2. EMBASE (via EMBASE.com) search strategy

('breast cancer'/exp OR 'Breast Neoplasm':ab,ti OR 'Neoplasm, Breast':ab,ti OR 'Breast Tumors':ab,ti OR 'Breast Tumor':ab,ti OR 'Tumor, Breast':ab,ti OR 'Tumors, Breast':ab,ti OR 'Neoplasms, Breast':ab,ti OR 'Breast Carcinoma':ab,ti OR 'Breast Carcinomas':ab,ti OR 'Carcinoma, Breast':ab,ti OR 'Carcinomas, Breast':ab,ti OR 'Mammary Neoplasms, Human':ab,ti OR 'Human Mammary Neoplasm':ab,ti OR

‘Human Mammary Neoplasms’ ab,ti OR ‘Neoplasm, Human Mammary’:ab,ti OR ‘Neoplasms, Human Mammary’:ab,ti OR ‘Mammary Neoplasm, Human’:ab,ti OR ‘Breast Cancer’:ab,ti OR ‘Cancer, Breast’:ab,ti OR ‘Mammary Cancer’:ab,ti OR ‘Cancer, Mammary’:ab,ti OR ‘Cancers, Mammary’:ab,ti OR ‘Mammary Cancers’:ab,ti OR ‘Malignant Neoplasm of Breast’:ab,ti OR ‘Breast Malignant Neoplasm’:ab,ti OR ‘Breast Malignant Neoplasms’:ab,ti OR ‘Malignant Tumor of Breast’:ab,ti OR ‘Breast Malignant’:ab,ti OR ‘Breast Malignant Tumors’:ab,ti OR ‘Cancer of Breast’:ab,ti OR ‘Cancer of the Breast’:ab,ti) AND (‘Albumin Bound Paclitaxel’ OR ‘Paclitaxel, Albumin-Bound’ OR ‘Protein-Bound Paclitaxel’ OR ‘Paclitaxel, Protein-Bound’ OR ‘Protein Bound Paclitaxel’ OR Abraxane OR ‘ABI007’ OR ‘ABI-007’ OR ‘ABI 007’) AND (‘paclitaxel'/exp OR Anzatax OR ‘NSC-125973’ OR ‘NSC 125973’ OR ‘NSC125973’ OR Taxol OR ‘Taxol A’ OR ‘Bris Taxol’ OR ‘Taxol, Bris’ OR ‘Paclitaxel, (4 alpha)-Isomer’ OR ‘Paxene’ OR ‘Praxel’ OR ‘7-epi-Taxol’ OR ‘7 epi Taxol’ OR ‘Onxol’) AND (random*:ab,ti OR placebo*:de,ab,ti OR (double NEXT/1 blind*):ab,ti)

3. Cochrane search strategy

#1 MeSH descriptor: [Breast Neoplasms] explode all trees
#2 (breast):ti,ab,kw (Word variations have been searched)
#3 (neoplas*):ti,ab,kw (Word variations have been searched)
#4 (carcinoma*):ti,ab,kw (Word variations have been searched)
#5 (cancer):ti,ab,kw (Word variations have been searched)
#6 #3 OR #4 OR #5
#7 #2 AND #6
#8 #1 OR #7
#9 Albumin Bound Paclitaxel
#10 Paclitaxel
#11 #9 AND #10
#12 #8 AND #11

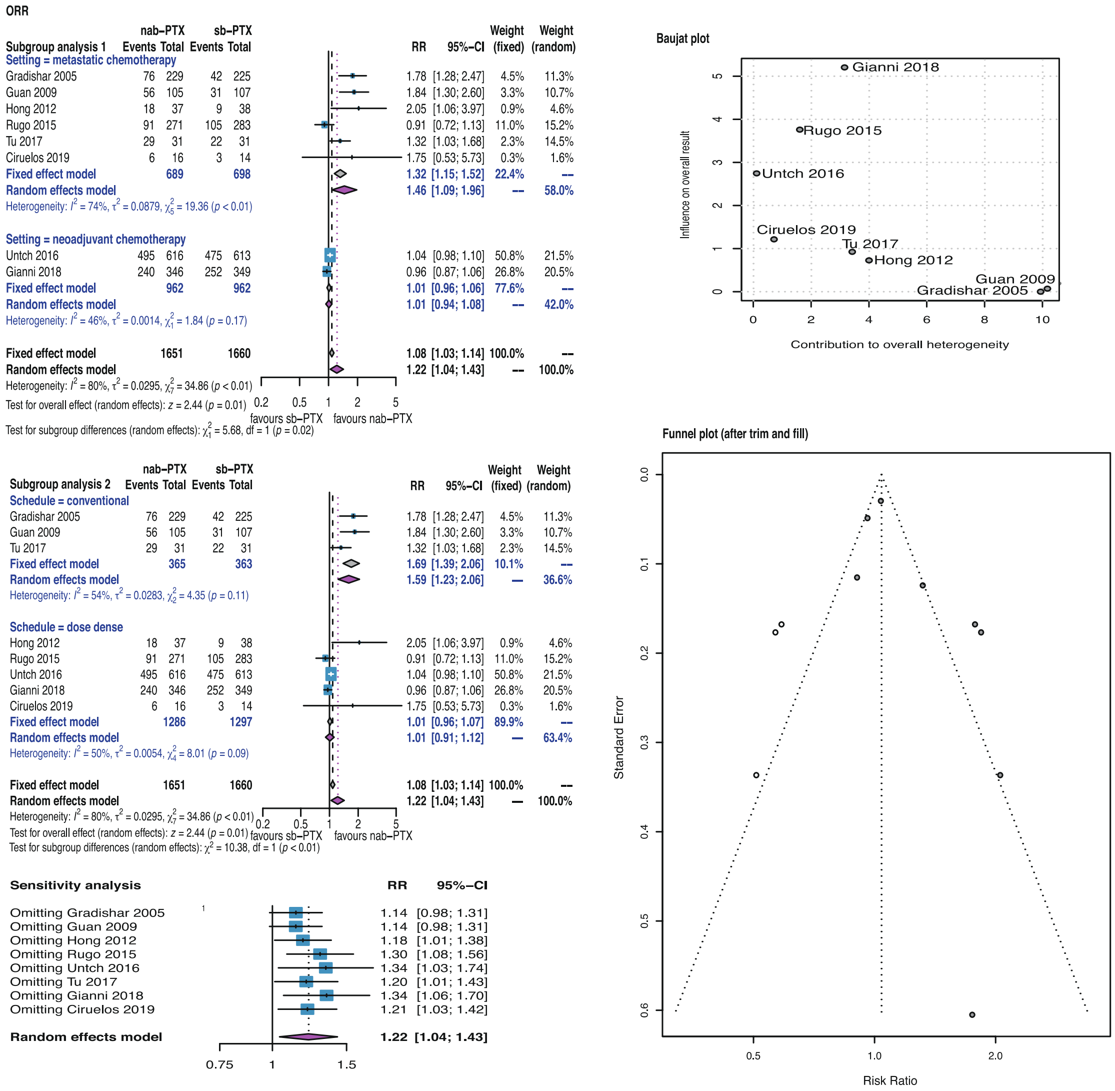

Figure S1 Subgroup analyses, sensitivity analysis and publication bias of objective response rate (ORR).

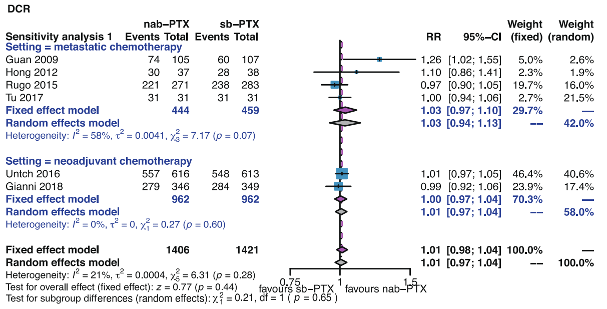

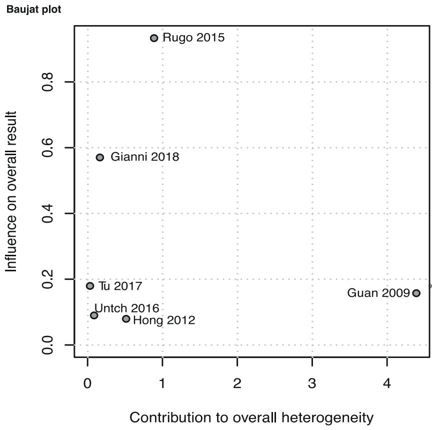

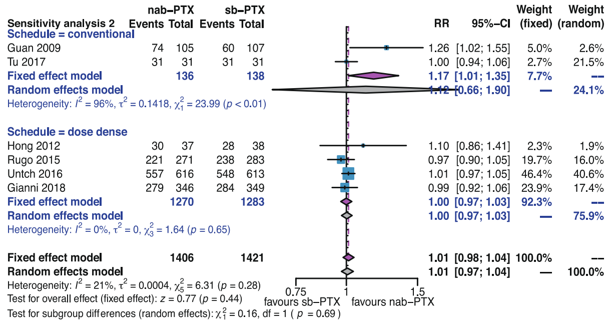

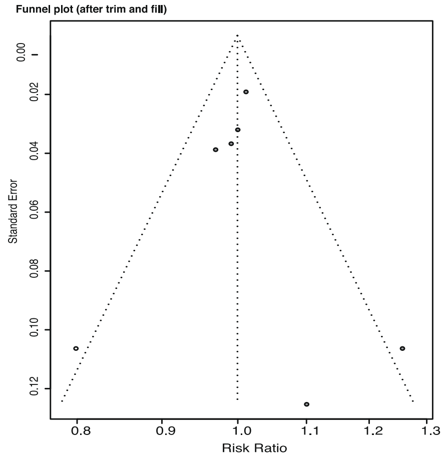

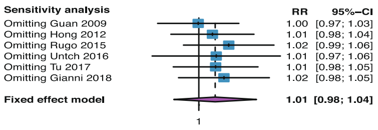

Figure S2 Subgroup analyses, sensitivity analysis and publication bias of disease control rate (DCR).

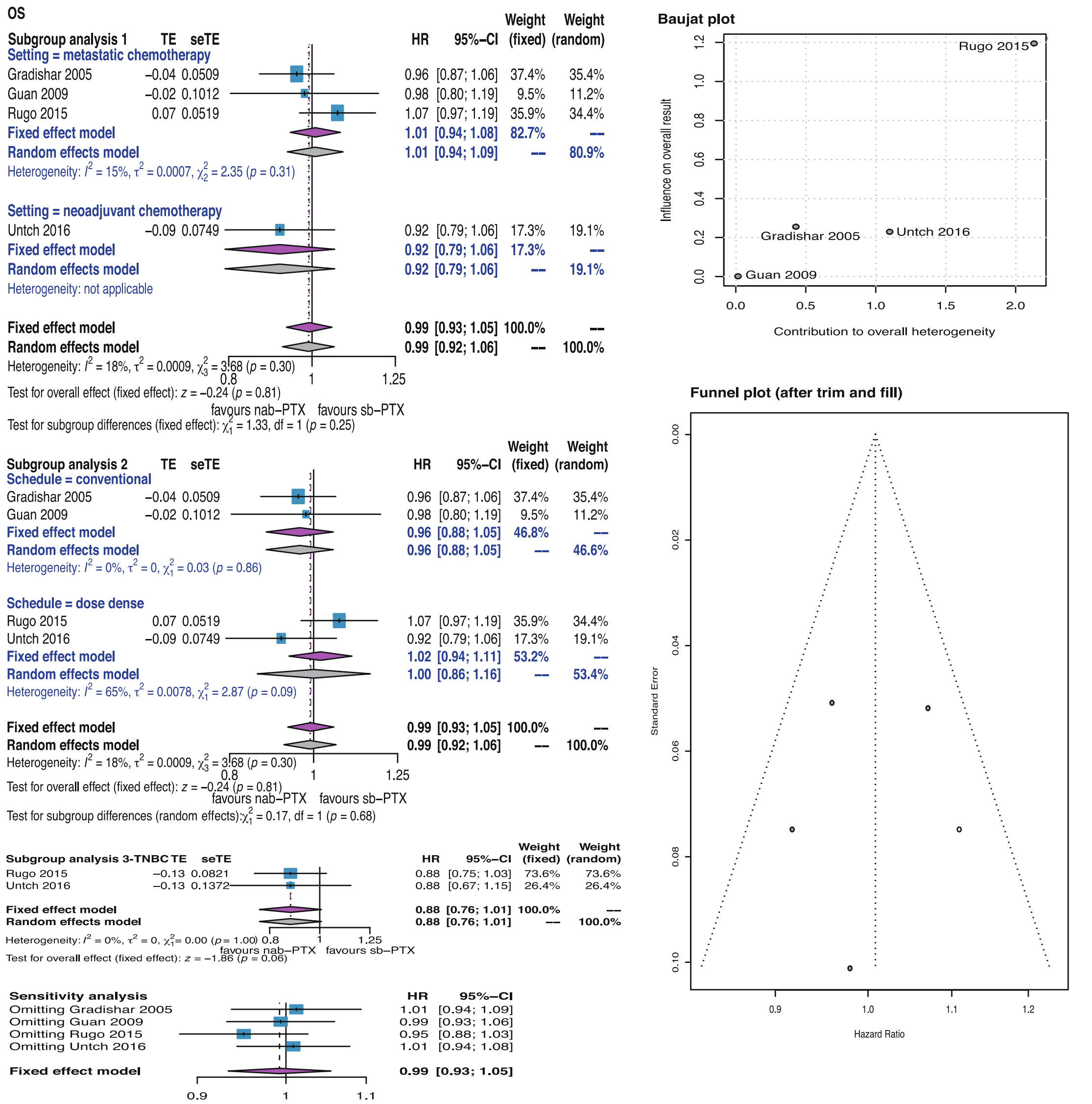

Figure S3 Subgroup analyses, sensitivity analysis and publication bias of overall survival (OS).

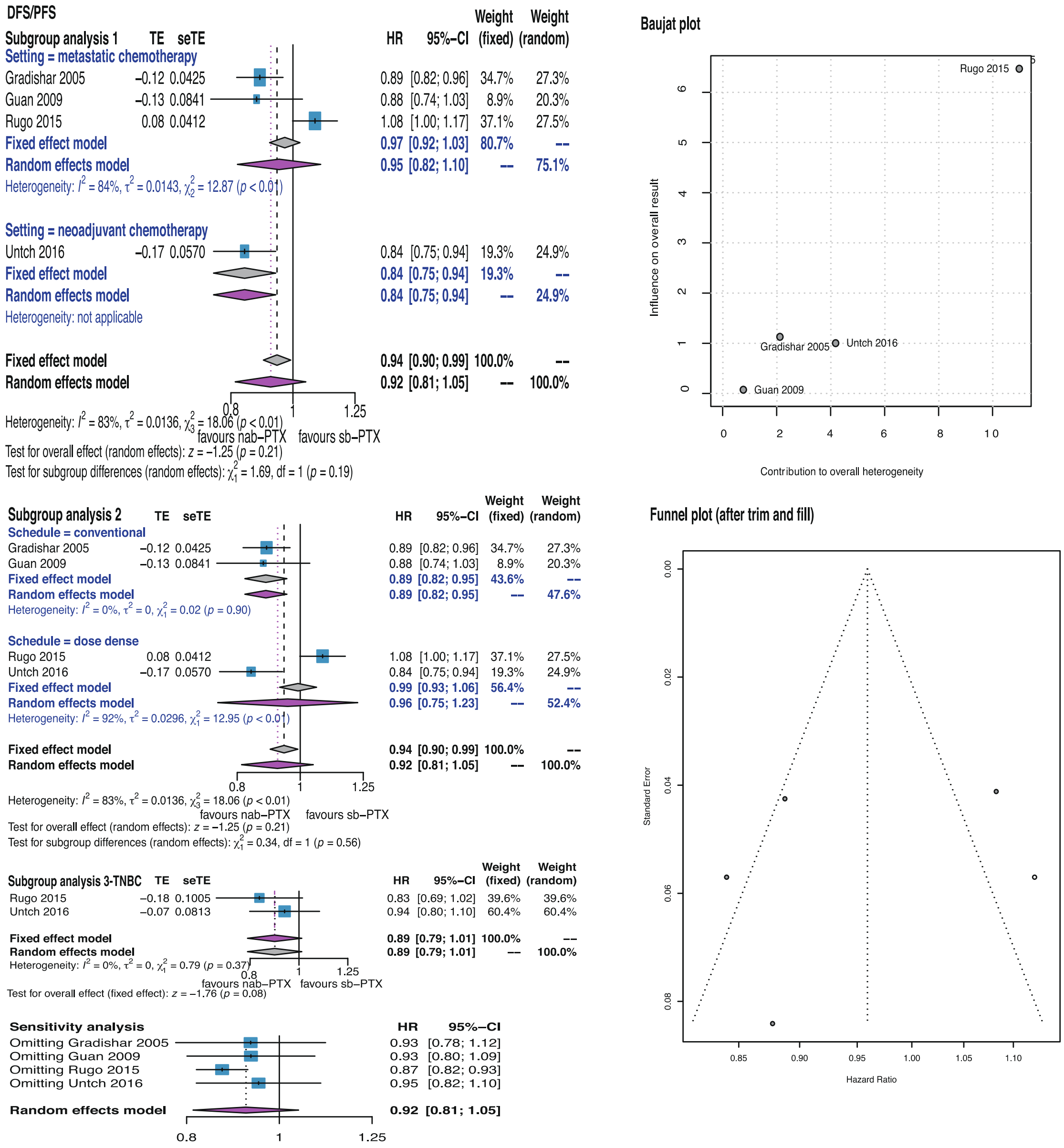

Figure S4 Subgroup analyses, sensitivity analysis and publication bias of disease-free survival/progression free survival (DFS/PFS).

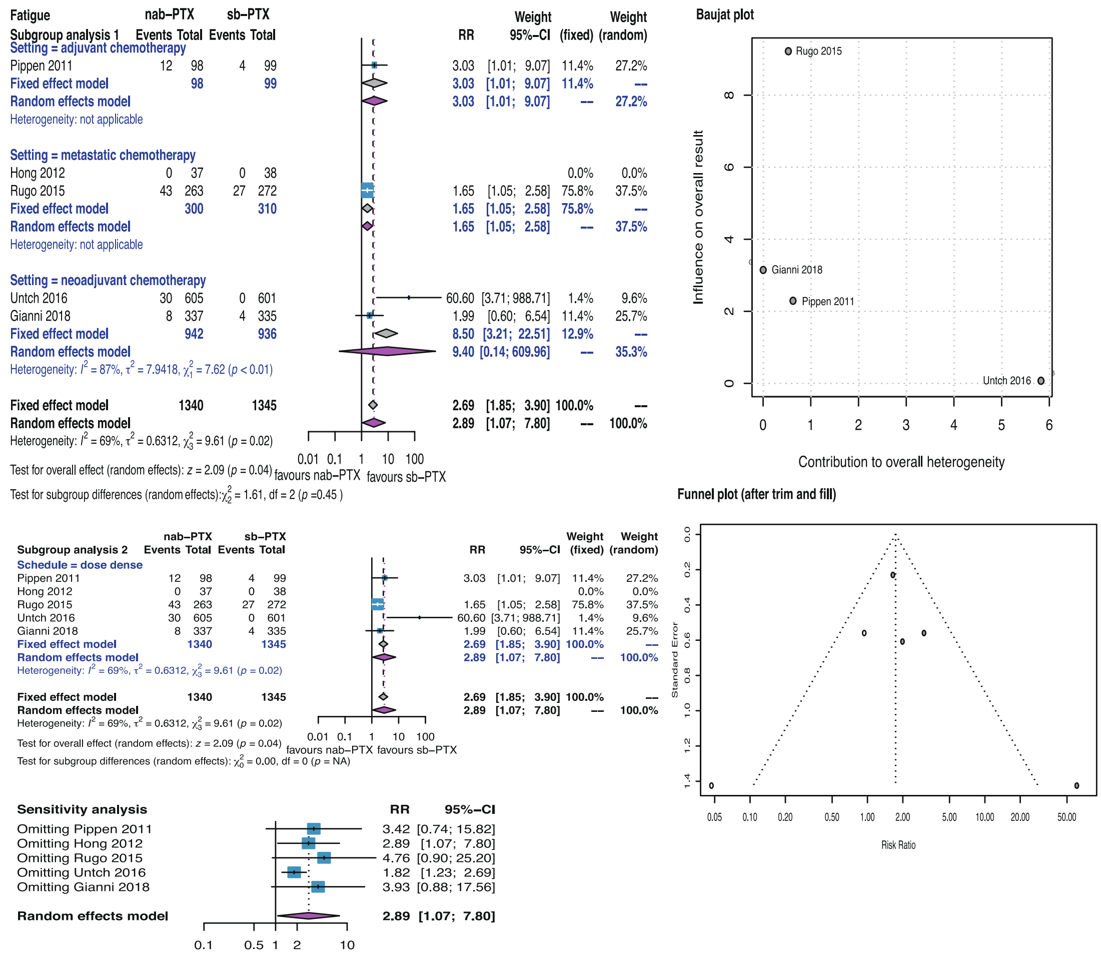

Figure S5 Subgroup analyses, sensitivity analysis and publication bias of fatigue.

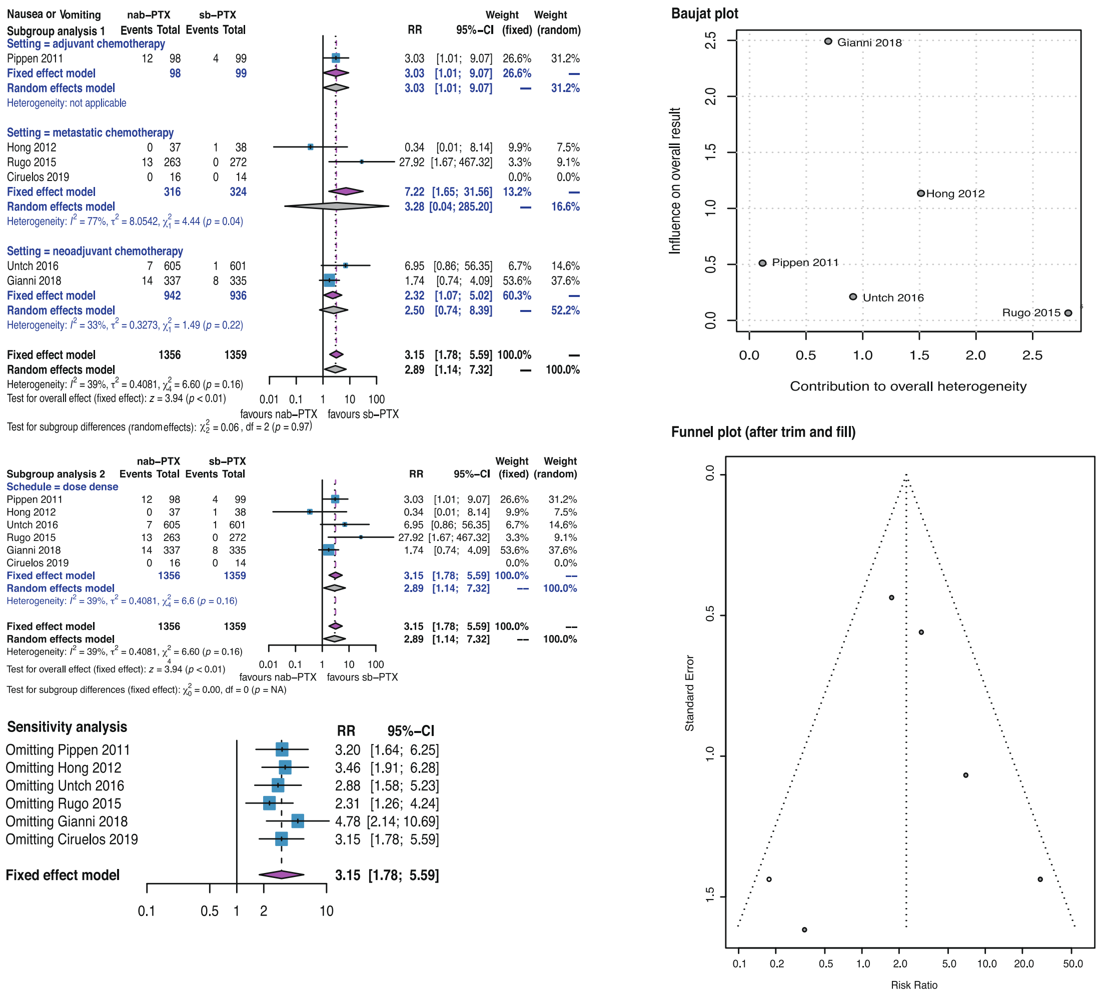

Figure S6 Subgroup analyses, sensitivity analysis and publication bias of nausea or vomiting.

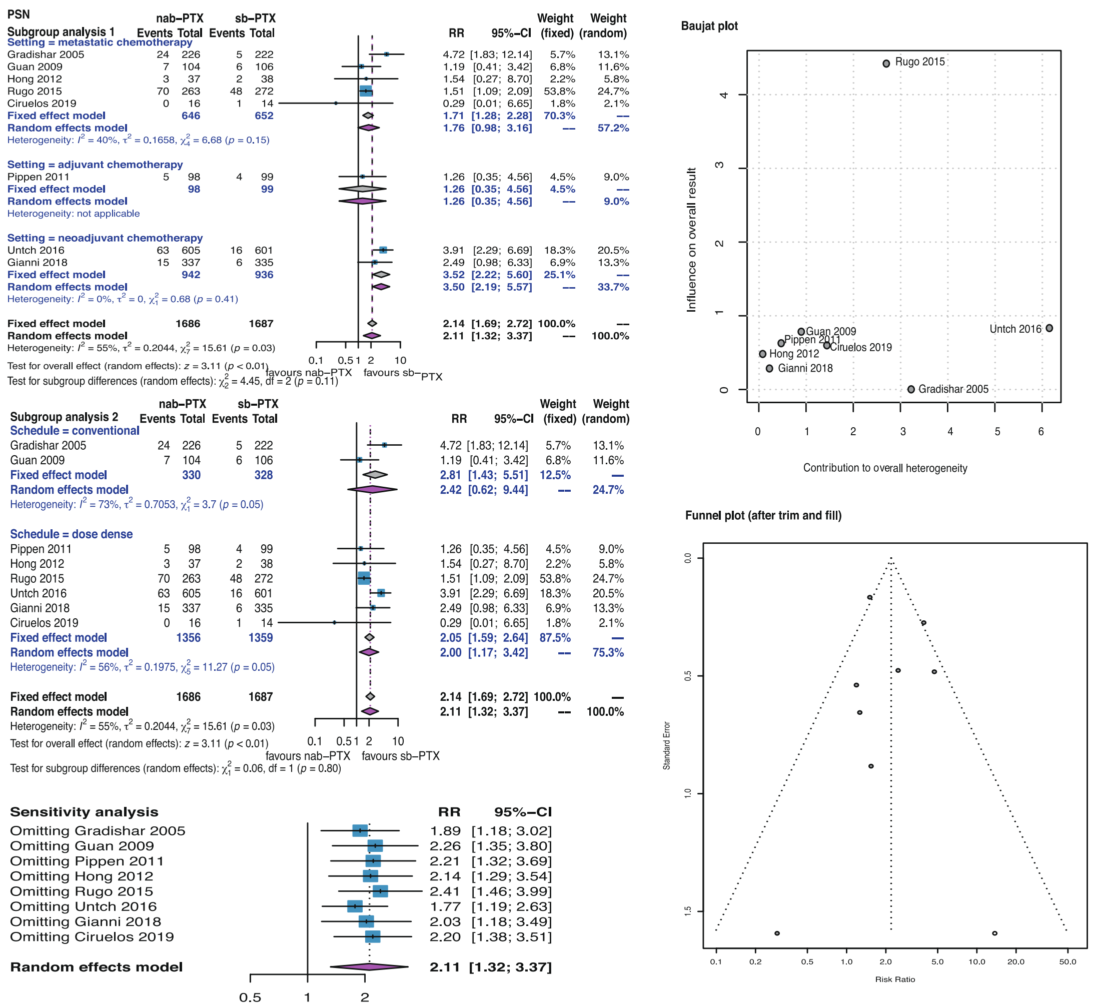

Figure S7 Subgroup analyses, sensitivity analysis and publication bias of peripheral sensory neuropathy (PSN).

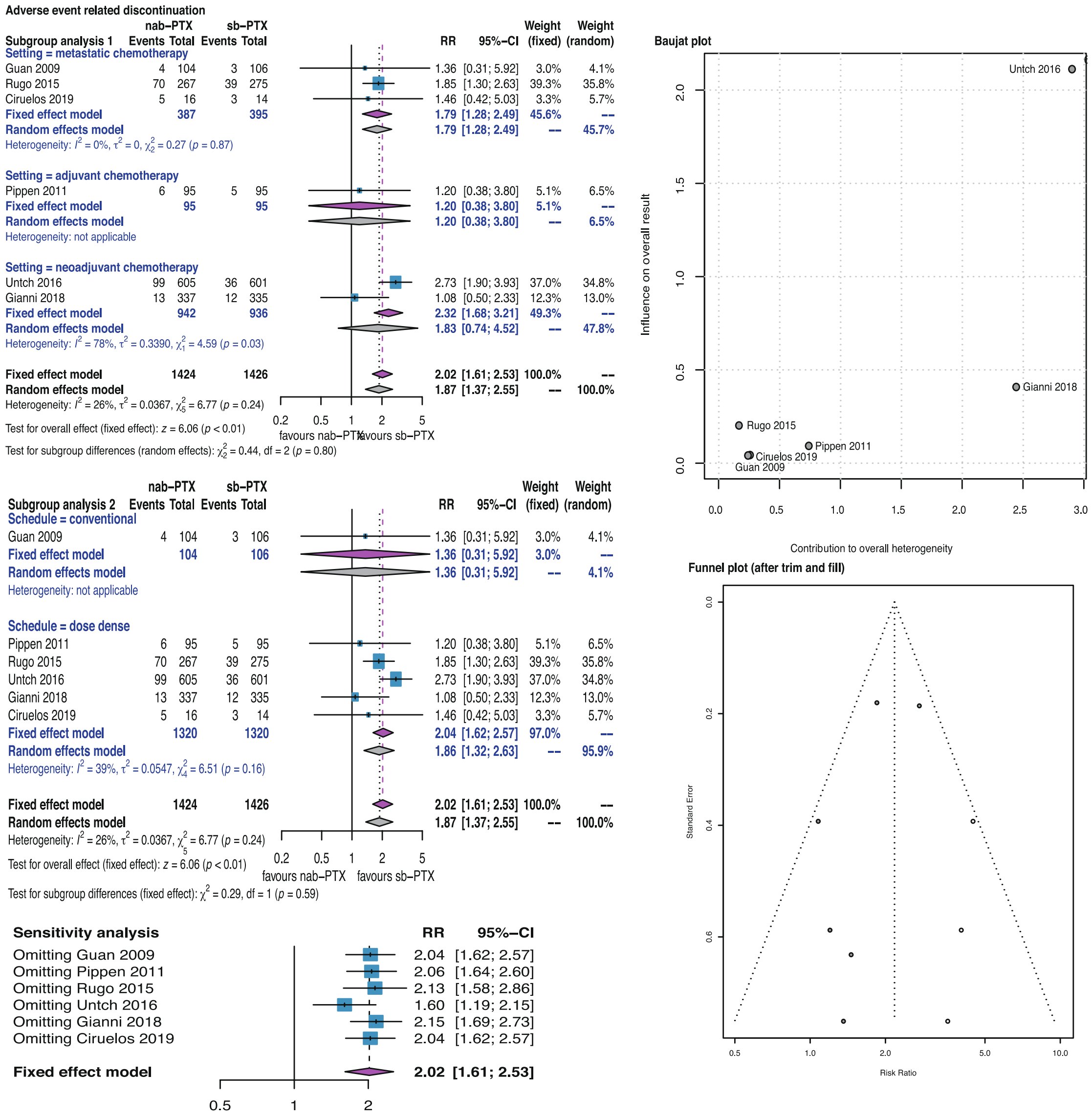

Figure S8 Subgroup analyses, sensitivity analysis and publication bias of adverse event related discontinuation (ARD).
